# Epidemic of 1853 in New Orleans

**Published:** 1854-02

**Authors:** 


### EPIDEMIC OF 1853 IN NEW ORLEANS.

THE REPORT of the "Howard Association of New Orleans," contains some instructive statistics in relation to the epidemic yellow fever which lately desolated that city. Thus, it appears that of 11,088 cases which came under the care of the Association, 5,845, or more than one-half, occurred in natives of Ireland, while only 716 were afforded by our native population. Among Germans there were 2,890 cases; 198 in natives of England; 56 Scotchmen; 436 Frenchmen, and small numbers among the natives of most other countries. Of the whole number 5,203 were males, and 5,885 were females; 9,415 were adults, and 1,673 under sixteen years of age. The number that died was 2,942, and of those discharged cured 8,146.

The amount of money disbursed by the Association was $159,-190 32, and the amount contributed $228,927 46, leaving a large balance in their hands for future charities. We remark that of this large sum expended, less than $15,000 went to Physicians for professional services, while the medicines cost over $19,000, and the amount paid for nursing patients at their residences was a little more than $25,120.

The Association give a most satisfactory account of their stewardship, and their Report will everywhere be read with interest. They say justly, that the example of fraternal fidelity and devotedness exhibited by our people on this memorable occasion is one which has few parallels in the history of men.

